# Pesticide Exposure among Latinx Children in Rural Farmworker and Urban Non-Farmworker Communities: Associations with Locality and Season

**DOI:** 10.3390/ijerph20095647

**Published:** 2023-04-26

**Authors:** Thomas A. Arcury, Haiying Chen, Sara A. Quandt, Jennifer W. Talton, Kim A. Anderson, Richard P. Scott, Phillip Summers, Paul J. Laurienti

**Affiliations:** 1 Department of Family and Community Medicine, and Center for Worker Health, Wake Forest University School of Medicine, Winston-Salem, NC 27157, USA; 2 Department of Biostatistics and Data Science, Division of Public Health Sciences, Wake Forest University School of Medicine, Winston-Salem, NC 27157, USA; 3 Department of Epidemiology and Prevention, Division of Public Health Sciences, and Center for Worker Health, Wake Forest University School of Medicine, Winston-Salem, NC 27157, USA; 4 Department of Environmental and Molecular Toxicology, Oregon State University, 2750 SW Campus Way, Corvallis, OR 97331, USA; 5 Department of Radiology, Wake Forest University School of Medicine, Winston-Salem, NC 27157, USA

**Keywords:** child health, pesticides, exposure assessment, personal monitoring, farmworkers, community-based participatory research, health equity

## Abstract

This study uses repeated measures to document the pesticide exposure of rural and urban Latinx children (age eight at baseline), and to compare these children in terms of the frequency and concentration of their exposure to a large set of pesticides, accounting for season. We used silicone wristbands worn for one week up to ten times at quarterly intervals from 2018 to 2022 to assess pesticide exposure in children from rural farmworker (*n* = 75) and urban non-farmworker (*n* = 61) families. We determined the detection and concentrations (ng/g) of 72 pesticides and pesticide degradation products in the wristbands using gas chromatography electron capture detection and gas chromatography mass spectrometry. The most frequently detected pesticide classes were organochlorines, pyrethroids, and organophosphates. Controlling for season, organochlorine or phenylpyrazole detections were less likely for rural children than for urban children. Detections of organochlorines, pyrethroids, or organophosphates were lower in spring and summer versus winter. Controlling for season, urban children had greater concentrations of organochlorines, while rural children had greater concentrations of pyrethroids and Chlorpyrifos. Pesticide concentrations were lower in winter and spring compared with summer and fall. These results further document that pesticides are ubiquitous in the living environment for children in vulnerable, immigrant communities.

## 1. Introduction

Pesticides include a wide variety of substances used to control pests. They include insecticides, herbicides, fungicides, and rodenticides, as well as additional formulations that target other organisms [1]. Pesticides have immediate and long-term consequences for human health [2]. Depending on dose, immediate consequences may include nausea, rash, burning eyes, muscle ache, coma, and death. Long-term consequences, even for repeated low-dose exposure, include increased risks for cancer, neurodegenerative disease, and reproductive problems [3,4,5,6].

The potential consequences of pesticide exposure for child health are greater than those for adult health. Children experience greater pesticide exposure than do adults due to a greater surface-to-volume ratio, being closer to the ground, having greater hand-to-mouth behaviors, and being less mature decision makers. Once exposed, children do not metabolize pesticides as efficiently as adults due to less mature metabolic processes. Children have immature and developing reproductive and neurological systems, and pesticide exposure can disrupt development of these systems. Children have greater life expectancy than adults, and so have more years in which the effects of pesticide exposure can manifest [7,8,9,10,11].

All United States (US) residents, not only those working or living in agricultural communities, continuously experience pesticide exposure [12,13,14]. US residents who are members of minority groups or otherwise live in vulnerable communities may experience greater pesticide exposure than the remainder of the population. The housing and neighborhoods in which they live are often old, in poor repair, and crowded. These characteristics increase the risk of pest infestation and pesticide use. Pesticides applied in the past may remain in these older environments. Finally, members of these groups often work in manual occupations that expose individual workers to chemicals such as pesticides and increase the risk of their families’ exposure through take-home pathways [12,13,14,15].

Latinx constitute a large and growing vulnerable minority population in the US. They are also a group for whom pesticide exposure is a particular concern due to their frequent employment in agriculture, landscape and lawn maintenance, building maintenance and cleaning, and construction. Pesticide-exposure research with Latinx communities has focused on adults and children in agricultural communities, particularly those in farmworker families [16,17,18,19,20,21,22,23,24,25]. However, pesticides are used in many industries, and are applied for residential and community pest control. Some research indicates that Latinx children in non-agricultural communities experience substantial pesticide exposure [26,27]. Pesticide urinary metabolites are highly prevalent among Latinx adult women and men living in agricultural as well as urban communities [28,29].

Research on Latinx child pesticide exposure has focused largely on organochlorine and organophosphate pesticides, both of which are neurotoxicants. The US has largely banned the use of organochlorine pesticides. However, being persistent, they remain in the environment [16,17,18,30,31]. The US has also banned the use of many organophosphate pesticides for residential and agricultural applications. Organophosphate pesticides are non-persistent and break down when exposed to sunlight [32], but they often remain in protected environments (e.g., inside buildings) for years [15,33,34]. Latinx or other children’s exposure to other insecticides, such as pyrethroids, is not well documented [17,23,25]. Information about child exposure to other pesticides, such as fungicides and herbicides, remains limited. We do not know the actual pesticides to which Latinx children, as well as other minority and vulnerable children, are exposed, nor the levels of their exposure.

In an earlier paper, we compared the pesticide exposure of Latinx children residing in rural agricultural communities with that of those living in urban non-agricultural communities [35]. These data were limited to the baseline contact with these children in 2018–2019, when they were eight years old. We found that these children experienced exposure to multiple pesticides. On average, each child was exposed to 2.7 different pesticide classes, and to 5.7 different specific pesticides. The most frequently detected pesticide classes were organochlorines, detected for 85.7% of the children, pyrethroids, detected for 65.4% of the children, and organophosphates, detected for 59.4% of the children. The most frequently detected specific pesticides were alpha-Chlordane (69.2%), trans-Nonachlor (67.7%), gamma-Chlordane (66.2%), Chlorpyrifos (54.9%), Cypermethrin (49.6%), and trans-Permethrin (39.1%). Rural and urban children differed in terms of the detected pesticides. More of the urban children had detections of organochlorines (93.3% vs. 79.5, *p* = 0.0228) and pyrethroids (75.0% vs. 57.5%, *p* = 0.0351) than did the rural children; more rural children had detections for organophosphates (71.2% vs. 45.0%, *p* = 0.0022). Urban children had greater concentrations of alpha-Chlordane (geometric mean [GM] 18.98, 95% confidence interval [CI] 14.14, 25.47 vs. 10.25, 95% CI 7.49, 14.03; *p* = 0.0055) and Dieldrin (GM 17.38, 95% CI 12.78 23.62 vs. 8.10, 95% CI 5.47, 12.00; *p* = 0.0034) than did rural children. These results were limited by differences in the seasons in which we collected the pesticide-exposure data; we assessed more of the urban than the rural children in the spring and summer.

Scarce research has examined urban–rural differences in child pesticide exposure, and no research has considered seasonal differences in child pesticide exposure. In this analysis, we use data collected over five years, 2018 to 2022, in which we assessed the pesticide exposure of the participating children up to 10 times at approximately quarterly intervals. Our aims for this analysis are to document the pesticide exposure of rural and urban Latinx children, and to compare the frequency and concentration of exposure to a large set of pesticides for rural and urban children (age eight years at baseline), accounting for season in which we assessed the children.

## 2. Materials and Methods

### 2.1. Overview—The PACE5 Study

This analysis uses repeated measures data from Preventing Agricultural Chemical Exposure 5 (PACE5) [35], a community-based participatory research project conducted in partnership between the North Carolina Farmworkers Project (Benson, NC, USA; https://ncfwp.org/ accessed on 2 February 2023) and Wake Forest University School of Medicine. PACE5 is a large, two-group, prospective study examining the health and cognitive effects of pesticide exposure for children in rural, Latinx farmworker families (rural children) and in similar urban, Latinx families (urban children) that do not include members employed as farmworkers. The PACE5 protocol and procedures have been approved by the Wake Forest University School of Medicine Institutional Review Board.

### 2.2. Participants

The PACE5 rural children resided in eastern North Carolina, in the counties surrounding the town of Benson. The PACE5 urban children resided in central North Carolina, in the counties surrounding the city of Winston-Salem.

Inclusion criteria: Inclusion criteria for rural and urban children were similar. Children were aged eight years at baseline and had completed first grade in the US. The eight-year age criterion was based on issues surrounding brain anatomy, and brain and cognitive development. Brain anatomy in regions consistent with the default mode network (DMN) is sensitive to pesticide exposure [36]. The DMN develops later in childhood and shows peak gray matter thickness between eight and ten years of age [37,38]. Children’s cognitive performance is associated with the timing of cortical development in key components of the DMN. Children with peak cortical thickness occurring between 9 and 12 years of age have the highest cognitive function [39]. Given this range of brain and cognitive development, the study was designed to recruit children when they were eight years old and longitudinal measurements were performed until they were ten years old.

Children were from families that self-identified as Latino or Hispanic with household incomes below 200% of the US federal poverty line. For inclusion, a parent of the rural children, or a parent’s co-resident partner, had to have been employed in farm work on non-organic farms during the past three years. Adults (parents, partners, other adults) living in the same house as the urban children had not been employed in an industry that involved routine exposure to pesticides (e.g., farm work, landscaping, pest control) in the previous three years. The urban children had not lived adjacent to agricultural fields in the previous three years. We excluded children from the study if they had a life-threatening illness, prior history of neurological conditions, or a physical condition or developmental disorder that would not allow them to complete or would interfere with the results of neurobehavioral tests or magnetic resonance imaging (MRI) (used in the larger main study). Children were excluded if a primary language other than Spanish or English was spoken in the home, or if a parent/guardian refused to complete the questionnaires.

Recruitment and informed consent: From March 2018 to December 2019, the North Carolina Farmworkers Project and other community organizations serving farmworkers’ families developed a list of rural families with an eight-year-old child, and the locations where they lived. These included families who had participated in a previous study of child growth and nutrition [40]. Local recruiters and community members in Winston-Salem developed a similar list of families that had an eight-year-old child. Bilingual staff members contacted the rural and urban parents, explained the overall study procedures, and answered questions. If the parent agreed to participate, they obtained signed informed consent from the parent and assent from the child. We do not know the number of potential participants or their parents who refused to participate because interviewers worked through community partners.

Sample size: The initial sample included 76 rural children and 65 urban children. We included in this analysis 75 rural children and 61 urban children who completed at least one pesticide-exposure assessment. The rural children completed 695 pesticide-exposure assessments and the urban children completed 473 pesticide-exposure assessments over up to ten follow-ups, to provide a total of 1168 pesticide-exposure assessments.

### 2.3. Data Collection

Schedule: The planned PACE5 data collection schedule included a baseline parent interview and an associated pesticide-exposure assessment using a silicone wristband worn by the child for one week. We then planned to complete eight follow-up interviews and wristband pesticide-exposure assessments at three-month intervals over two years. The COVID-19 pandemic affected this data-collection schedule. We paused data collection for three months in March to May 2020. Some breaks between data points were greater than planned (some breaks were up to 13 months rather than 3 months). The total period over which we collected data was longer than planned. We recruited participants from March 2018 to December 2019; therefore, data collection should have ended in January 2021. However, due to the break, we did not complete data collection until March 2022.

Questionnaires: The baseline and subsequent questionnaires included items to measure demographic and background characteristics of the family and child. We adapted available Spanish-language items and scales from existing questionnaires when available [41]. We developed new items in English, had them translated into Spanish by a native Spanish speaker, and back translated into English by a native English speaker. Our community partners reviewed the questionnaire content and items. Seven Spanish-speaking individuals from rural and urban communities completed pre-test interviews. We revised questionnaire items based on the review and pre-testing.

Pesticide-exposure assessment: We purchased the wristbands from 24hourwristbands (Houston, TX). We previously described the conditioning, post-deployment cleaning, and extraction of the wristbands [42,43,44]. Briefly, we thermally conditioned the wristbands in a Blue-M Model# POM-18VC-2 vacuum oven (300 °C, 0.12 Torr) for three hours with a periodic N_2_ sweep [43]. Post-conditioning, we packaged wristbands in polytetrafluoroethylene (PTFE) bags prior to deployment.

Procedures: Interviewers were native Spanish speakers; all spoke English, but with varying degrees of proficiency. We trained the interviewers regarding participant inclusion criteria, recruitment procedures, questionnaire content, and collecting the silicone wristbands. The interviewers completed audio-recorded practice interviews before the start of data collection. The interviewers entered data in real time using Research Electronic Data Capture (REDCap), hosted at Wake Forest University School of Medicine through the Clinical and Translational Science Institute [45].

At the end of the interview, interviewers gave the child a silicone wristband for passive pesticide-exposure assessment. The interviewers gave the parent and child specific instructions on how the child should wear the wristband on seven consecutive days [43]. The interviewers also gave the parent and child a Teflon bag for storing the wristband at the end of the seven-day period. The interviewers scheduled a time to retrieve the wristband, and stored it in the Teflon bag throughout shipment to the laboratory. The parent and child received a USD 20 incentive for completing the questionnaire and a USD 10 incentive for wearing the silicone wristband at the baseline and at each follow-up.

### 2.4. Laboratory Analysis

At post-deployment cleaning, we rinsed the wristbands with 18 MΩ·cm water and isopropanol to remove particulate matter. We added extraction surrogates tetrachloro-meta-xylene (TCMX), decachlorobiphenyl, and PCB100, and we extracted the wristbands with two 50 mL volumes of ethyl acetate, combined and quantitatively reduced to 1 mL. To remove analytical interferences, a 200 μL aliquot of extract underwent solid-phase extraction (SPE) on a C18 silica column with acetonitrile [43,45], followed by a solvent exchange to isooctane for injection. All solvents were Optima-grade or equivalent (Fisher Scientific, Pittsburgh, PA, USA). All analytical grade standards were purchased from Accustandard (New Haven, CT, USA) with purities of 95% or higher.

Analysis of pesticides in the wristbands used a validated 22-min gas chromatograph with dual micro-electron detectors (GC-ECD) [23,35,46,47,48]. We added 4,4′-dibromooctafluorobipheny as an internal standard, and we analyzed extracts using an Agilent 6890N GC-ECD. We performed simultaneous injection using dual 7683 auto samplers onto DB-17MS and a DB-5MS column. A complete analyte list, detection limits, quantitation limits, and chromatographic conditions are presented in the electronic Supplementary Material (Supplemental Tables S1 and S2). We identified and quantified most analytes on the DB-17MS column, with the DB-XLB column used for confirmation.

Quality assurance/quality control: We performed sample handling, analysis, and quantitation as defined by laboratory data quality objectives and standard operating procedures. Surrogate standard compounds accounted for any loss during extraction and analysis of passive sampling devices. We analyzed instrument blanks and performed regular continual calibration checks during chromatography. We included construction and process blanks in the analysis.

### 2.5. Measures

Personal characteristics at baseline: Locality was the basic characteristic for each child and was categorized as rural (farmworker family) versus urban (non-farmworker family), as defined by the inclusion criteria. Other characteristics included the child’s gender (girl or boy as stated by a parent), whether the child was born in the US or another country (e.g., Mexico, Guatemala), whether both parents were present in the household, and the number of people in the household, with a value of 3–4, 5–6, or 7 or more.

Season: We classified measures of pesticide detections and concentrations according to the season in which we collected the samples (the season in which the participant wore the wristband). We defined winter as those samples collected in December to February, spring as those collected in March to May, summer as those collected in June to August, and fall as those collected in September to November.

Pesticide exposure: Specific pesticide-detection measures were the wristband detections (presence/absence) of each of 72 specific pesticides and pesticide degradation products. We based the selection of pesticides and pesticide degradation products on toxicology and exposure studies [43] and previous studies [23], coupled with method-validation studies for the wristbands and the analytical method. Pesticide class detection measures were detections (presence/absence) of at least one specific pesticide from each of 15 pesticide classes: organochlorine, pyrethroid, organophosphate, phenylpyrazole, neonicatinoid, chloroneb, dicarboximide, pentachloronitrobenzene, thiadiazole, dinitroaniline, aniline, triazine, benzenedicarboxylic acid, oxadiazole, and thiocarbamate. Total pesticide classes detected refers to the number of different pesticide classes (0 to 15) detected in a wristband. Total specific pesticide detections indicate the number of different specific pesticides (0 to 72) detected in a wristband. The number of organochlorine detections refers to the number of different organochlorine pesticides detected in a wristband (0 to 32), and number of pyrethroid detections reports the number of different pyrethroid pesticides detected in a wristband (0 to 8). We did not calculate this measure for the other pesticide classes because usually only one pesticide from these classes was detected in the wristbands. Pesticide concentrations are reported in ng/g as GMs. We limited the calculation of concentrations to those samples in which we detected a specific pesticide [35,48]. We calculated concentrations only for those pesticides with detections in at least 10% of the overall total wristbands, for both rural and urban localities, and in wristbands from each of the four seasons.

### 2.6. Statistical Analysis

We first examined the collection of wristband assessments across the different time points and the different seasons. We used descriptive statistics, counts, and percentages for binary variables along with means and standard deviations (SD) for continuous variables to summarize the children’s baseline characteristics and the distribution of samples of various specific pesticides, pesticide degradation products, and pesticide classes. For outcomes with sufficient detections, we used a generalized estimating equation (GEE) method to evaluate the effect of locality and season on the presence of detection while accounting for repeated measures for the same child. We report odds ratios (OR) and corresponding 95% CI. Additionally, we fit general linear mixed-effects models (LMM) to test the concentration differences between the two localities and four seasons for values above the limit of detection. We applied log transformation to achieve better normality and we back transformed the estimates to obtain GMs and 95% CIs for concentrations. We analyzed total pesticide class detections, total specific pesticide detections, numbers of organochlorine detections, and numbers of pyrethroid detections in similar LMMs. We tested locality by seasonal interaction in all models. We considered a *p* value of <0.05 to be statistically significant.

## 3. Results

### 3.1. Child Characteristics

Based on the inclusion criteria, all children were 8 years old at baseline, all were Latinx, all had completed the first grade, and all were in families with incomes below 200% of the poverty line. This analysis included 75 rural children and 61 urban children (Table 1). The children were evenly divided by gender, with 68 girls and 68 boys. Most (91.1%) children were born in the US. Most (86.6%) children lived in families in which both of their parents were present. One-third (32.3%) of the children lived in families of three or four people, 51.5% lived in families of five or six people, and 16.2% lived in families of seven or more.

### 3.2. Data Characteristics

Rural children completed 695 wristband pesticide assessments and urban children completed 473 assessments, providing 1168 total assessments (Table 2). Children completed the fewest assessments in spring (235 [20.12%] total, 154 rural, 81 urban), with 296 (25.3%) assessments completed in winter, 330 (28.3%) completed in summer, and 307 (26.3%) completed in fall. Of the 136 children, 118 (86.8%) completed at least one assessment in all four seasons. Altogether, 71 (94.7%) of the rural children and 47 (77.0%) of the urban children completed at least one assessment in all four seasons. In total, 86 (63.2%) children completed 10 assessments, with 55 (73.3%) rural and 31 (50.8%) urban children completing 10 assessments.

### 3.3. Specific Pesticide Detections and Concentrations

We detected 62 of 72 pesticides or pesticide degradation products included in the laboratory analysis (Table 3). Insecticides were the most commonly detected pesticides. We detected4 (of 25) organochlorine insecticides in at least 10% of the total samples by rural–urban locality and by season. These included alpha-Chlordane (71.8% of total samples), gamma-Chlordane (74.8%), Dieldrin (28.1%), and trans-Nonachlor (70.6%). We detected three (of eight) pyrethroid insecticides in at least 10% of the samples, including Cypermethrin (65.2%), cis-Permethrin (48.3%), and trans-Permethrin (49.5%). We detected 1 (of 12) organophosphate insecticide, Chlorpyrifos (56.9%), in at least 10% of the samples. We detected one (of three) phenylpyrazole insecticides, Fipronil-sulfide (12.9%), and the one aromatic fungicide, Chloroneb (12.9%), in at least 10% of the samples.

We focused analysis on ten commonly detected pesticides (alpha-Chlordane, gamma-Chlordane, Dieldrin, trans-Nonachlor, Cypermethrin, cis-Permethrin, trans-Permethrin, Chlorpyrifos, Fipronil-sulfide, and Chloroneb) that were detected in at least 10% of the total detections, as well as by locality and season (Table 4). Rural children had only about one-quarter the likelihood of detection of the four organochlorines, alpha-Chlordane (OR 0.26, 95% CI 0.17, 0.41), gamma-Chlordane (OR 0.27, 95% CI 0.17, 0.42), Dieldrin (OR 0.29, 95% CI 0.19, 0.44), and trans-Nonachlor (OR 0.29, 95% CI 0.18, 0.45), compared with urban children. Rural children had only about one-half the likelihood of detection of the Phenylpyrazole, Fipronil-sulfide (OR 0.54, 95% CI 0.36, 0.81). Rural children had over twice the likelihood (OR 2.43, 95% CI 1.62, 3.62) of detection of the organophosphate Chlorpyrifos compared with urban children. Children did not differ by locality in the detection of the three pyrethroids, Cypermethrin, cis-Permethrin, and trans-Permethrin, or the aromatic fungicide, Chloroneb.

The likelihood of detection of alpha-Chlordane (OR 0.38, 95% CI 0.26, 057), gamma-Chlordane (OR 0.37, 95% CI 0.25, 0.55), trans-Nonachlor (OR 0.48, 95% CI 0.33, 0.68), Cypermethrin (OR 0.53, 95% CI 0.39, 0.72), cis-Permethrin (OR 0.56, 95% CI 0.43, 0.74), trans-Permethrin (OR 0.59, 95% CI 0.44, 0.78), and Chlorpyrifos (OR 0.63, 95% CI 0.46, 0.86) was significantly lower in the spring compared with the winter. This was not true for Dieldrin (OR 0.79, 95% CI 0.55, 1.14), Fipronil-sulfide (OR 0.98, 95% CI 0.58, 1.65), or Chloroneb (OR 0.67, 95% CI 0.37, 1.22). The likelihood of detection of alpha-Chlordane (OR 0.35, 95% CI 0.24, 051), gamma-Chlordane (OR 0.45, 95% CI 0.31, 0.66), Dieldrin (OR 0.67, 95% CI 0.47, 0.96), trans-Nonachlor (OR 0.39, 95% CI 0.27, 0.57), Cypermethrin (OR 0.65, 95% CI 0.49, 0.87), and Chlorpyrifos (OR 0.61, 95% CI 0.47, 0.79), but not for cis-Permethrin (OR 0.76, 95% CI 0.57, 1.01), or trans-Permethrin (OR 0.82, 95% CI 0.62, 1.09) was significantly lower in the summer than in the winter. The likelihood of detection of alpha-Chlordane (OR 0.62, 95% CI 0.44, 0.89) was significantly lower in fall versus winter, with the likelihood of detection of Fipronil-sulfide being greater in summer versus winter (OR 1.71, 95% CI 1.09, 2.70). Detection of Chloroneb did not differ by season.

We found significant interactions between locality and season for four of the pesticides (Table 5). These largely amplified the associations already noted. In every season, rural children were less likely than urban children to have had trans-Nonachlor detected and this likelihood was lowest in spring. Rural children were more likely than urban children to have had cis-Permethrin or trans-Permethrin detected in fall or winter, and this was less likely to have been detected in spring or summer. Rural children were more likely than urban children to have had Fipronil-sulfide detected in spring, and less likely to have had this detected in summer, winter, or fall.

Urban children had significantly greater concentrations of organochlorine pesticides than did rural children (Table 6). The GM of alpha-Chlordane for urban children was 13.02 ng/g (95% CI 10.48, 16.18) compared with 7.99 ng/g (95% CI 6.56, 9.73) for rural children. Concentrations of gamma-Chlordane (GM 12.46 ng/g, 95% CI 9.96, 15.58 versus GM 7.14 ng/g, 95% CI 5.84, 8.74), Dieldrin (GM 12.97 ng/g, 95% CI 10.46, 16.08 versus GM 7.16 ng/g, 95% CI 5.78, 8.86), and trans-Nonachlor (GM 5.79 ng/g, 95% CI 4.66, 7.20 versus GM 3.84 ng/g, 95% CI 3.16, 4.67) were all significantly greater for urban versus rural children.

We found significantly greater concentrations of the three pyrethroid pesticides, Cypermethrin (GM 316.36 ng/g, 95% CI 244.39, 409.51 versus GM 129.73 ng/g, 95% CI 94.97, 177.19), cis-Permethrin (GM 162.68 ng/g, 95% CI 132.31, 200.02 versus GM 118.02, ng/g 95% CI 92.12, 151.22), and trans-Permethrin (GM 294.71 ng/g, 95% CI 240.55, 361.07 versus GM 213.18 ng/g, 95% CI 167.78, 270.86) among rural children compared with urban children. Rural children had significantly greater concentrations of the organophosphate pesticide Chlorpyrifos than did urban children (GM 12.91 ng/g, 95% CI 10.39, 16.04, versus GM 8.16 ng/g, 95% CI 6.20, 10.75).

For all of the organochlorine, pyrethroid, and organophosphate pesticides, we found the greatest seasonal concentrations to be in the summer, followed by the fall. For example, the GM for gamma-Chlordane was 10.53 ng/g (95% CI 8.85, 12.53) in the summer and 10.44 ng/g (95% CI 8.79, 12.40) in the fall, but 9.02 ng/g (95% CI 7.60, 10.72) in the winter and 7.98 ng/g (95% CI 6.61, 9.64) in the spring. The GM for Cypermethrin was 249.90 ng/g (95% CI 195.28, 319.79) in the summer and 218.17 ng/g (95% CI 171.50, 277.55) in the fall, but 196.67 ng/g (95% CI 154.42, 250.49) in the winter and 157.07 ng/g (95% CI 119.49, 206.47) in the spring. The GM for Chlorpyrifos was 11.98 ng/g (95% CI 9.76, 14.70) in the summer and 10.83 ng/g (95% CI 8.83, 13.28) in the fall, but 8.83 ng/g (95% CI 7.21, 10.80) in the winter and 9.70 ng/g (95% CI 7.80, 12.07) in the spring.

### 3.4. Pesticide Classes

We detected at least one organochlorine in 85.3% of the samples, at least one pyrethroid in 77.6% of the samples, at least one organophosphate in 58.9% of the samples, and at least one Phenylpyrazole in 15.8% of the samples (Table 7). We detected the fungicide Chloroneb in 12.1% of the samples. Rural children had a lower likelihood than urban children of having at least one organochlorine detected (OR 0.25, 95% CI 0.14, 0.43) (Table 8). Rural children had a greater likelihood than urban children of having at least one organophosphate detected (OR 2.21, 95% CI 1.49, 3.29). Rural children had a lower likelihood than urban children of having at least one phenylpyrazole detected (OR 0.60, 95% CI 0.42, 0.84). Rural and urban children did not differ in their comparative likelihoods of having Chloroneb or at least one pyrethroid detected.

The likelihood of a detection of at least one organochlorine (OR 0.52, 95% CI 0.33, 0.84), at least one pyrethroid (OR 0.49, 95% CI 0.34, 0.69), or at least one organophosphate (OR 0.69, 95% CI 0.51, 0.93) was lower in spring compared with winter. The likelihood of a detection of at least one organochlorine (OR 0.57, 95% CI 0.37, 0.88) or at least one organophosphate (OR 0.67, 95% CI 0.52, 0.88) was lower in summer compared with winter. The odds of at least one organochlorine, pyrethroid, or organophosphate detection did not differ between fall and winter. The likelihood of a detection of at least one phenylpyrazole (OR 1.76, 95% CI 1.15, 2.69) was greater in summer compared with winter. Chloroneb detection did not differ by season.

We found significant interactions between locality and season for one pesticide class (Table 9). Similar to the specific pesticide Fipronil-sulfide, rural children were more likely than urban children to have phenylpyrazole detected in spring, and less likely to have it detected in summer, winter, or fall.

On average, we detected 2.6 different pesticide classes and 5.8 different specific pesticides in each sample (Table 10). Total pesticide class detections did not differ by rural (2.6) and urban (2.7) locality. The average number of pesticide class detections was lower in spring (2.4), compared with summer (2.7), fall (2.7), or winter (2.7) (*p* = 0.0209). Total specific pesticide detections differed according to rural (5.4) or urban (6.3) locality (*p* = 0.0003). Total specific pesticide detections differed by season, with fewer classes detected in spring (5.3) and summer (5.6) compared with the winter (6.4) and fall (6.1) (*p* < 0.0001).

The average number of organochlorine detections was greater for urban (3.5) than for rural (2.5) children (*p* < 0.0001). The average number of pyrethroid detections did not differ by locality. Fewer average organochlorine detections occurred in the spring (2.7) and summer (2.7) compared with the winter (3.3) and fall (3.2) (*p* < 0.0001). Similarly, fewer average pyrethroid detections occurred in the spring (1.6) and summer (1.8) compared with the winter (2.0) and fall (1.9) (*p* < 0.0032).

## 4. Discussion

All of these Latinx children experience exposure to a large number of pesticides, and they experience these pesticide exposures repeatedly across the year. These pesticides include organochlorine, pyrethroid, organophosphate, and phenylpyrazole insecticides, as well as fungicides and herbicides. Pesticide exposure differs among these children by locality and by season. Over 80% of the samples indicated exposure to at least one organochlorine insecticide in each season of the year, with the mean number of specific organochlorine pesticide exposures in each season ranging from 2.5 to 3.3. At least 66% of the samples indicated at least one pyrethroid exposure and at least 54% indicated at least one organophosphate exposure in each season of the year. On average, each sample indicated exposure to at least five different pesticides in each season of the year, with the number of different pesticides greater in the fall and winter than in spring and summer. Our laboratory procedures included analysis of 72 pesticides and pesticide degradation products, and we detected 62 of these at least once across the 1168 samples. If we had been able to include laboratory analysis for additional pesticides, we would probably have documented that these children experienced exposure to an even greater number of specific pesticides. Our findings confirm that these children experience a substantial pesticide exposure burden.

This repeated measures analysis confirms the rural–urban pattern of pesticide detections reflected in the baseline data [35]. Most of these children, rural and urban, are exposed to organochlorine, pyrethroid, and organophosphate pesticides. More rural children are exposed to organophosphate pesticides, more urban children are exposed to organochlorine and phenylpyrazole pesticides, and rural and urban children are equally exposed to pyrethroid pesticides.

The repeated measures analysis expands the rural–urban pattern of pesticide wristband concentrations reported in our baseline data [35]. Organochlorine pesticide concentrations were greater for urban compared with rural children, according to this repeated measures analysis. This is similar to the baseline data. In the baseline data, we found no rural–urban differences in concentrations of pyrethroids. In the repeated measures analysis we found that rural children had significantly greater concentrations of three pyrethroid pesticides. Furthermore, in the baseline data, rural and urban children did not differ in terms of Chlorpyrifos concentration. Rural children in the repeated measures analysis had greater concentrations of Chlorpyrifos than did urban children. It appears that urban children have greater concentrations of organochlorine pesticides, which are historic pesticides remaining in the urban environment; rural children have greater concentrations of pyrethroid and organophosphate pesticides, which are contemporary pesticides widely used in agriculture.

We found season was associated with the percent of rural and urban children with pesticide detections. Among all children, fewer had detections in the spring and summer compared with the fall and winter. This pattern was unexpected. We expected that detections would be greater in spring and summer, particularly among those living in rural agricultural areas because that is when pesticides are applied to crops. We also found seasonal variations in pesticide concentrations. Among the organochlorine pesticides considered (alpha-Chlordane, gamma-Chlordane, Dieldrin, trans-Nonachlor), concentrations were greater in summer and fall compared with winter and spring. We found this same pattern of greater concentrations in summer and fall compared with winter spring for the three pyrethroids (Cypermethrin, cis-Permethrin, and trans-Permethrin) and one organophosphate (Chlorpyrifos) considered.

The patterns of lower spring-summer versus higher fall and winter detection levels suggest an explanation. Detection is lower in spring and summer because children spend less time indoors in their homes and in their schools. The older, substandard, and crowded houses in which many low-income Latinx families (rural and urban) live are reservoirs for an accumulation of pesticides applied or carried into homes (and schools) over years [15,33]. Because the buildings are older and crowded, and are often located in older, substandard, and crowded neighborhoods, they are more prone to pesticide use, and have had more pesticides applied over a long period. Because they are older, the pesticides that have been applied to them include those deemed more hazardous to human health and that have been banned from residential use or any use at all. These include organochlorine pesticides and most of the organophosphate pesticides (particularly Chlorpyrifos). Organochlorines are persistent pesticides that are expected to remain in buildings for years after they are applied [14]. Organophosphates and pyrethroids are non-persistent pesticides that are expected to degrade with time. However, with no exposure to UV light or water, organophosphates and pyrethroids may also remain in within the confines of buildings for decades.

The results of this analysis are similar to studies comparing rural and urban Latinx adult pesticide exposure [28,29,49]. These adult studies measured pesticide exposure using pesticide urinary metabolites and were focused on organophosphate and pyrethroid insecticides. Most of the rural and urban Latinx men and women in these studies experienced organophosphate and pyrethroid exposure. For example, in 2013, all of the rural farmworker and urban non-farmworker men were exposed to at least one organophosphate and 95% of those men were exposed to at least one pyrethroid pesticide [28]. Similarly, in 2013, all of the rural farmworker women and 96% of the urban non-farmworker women were exposed to at least one organophosphate, and 90% of both the rural farmworker and the urban non-farmworker women were exposed to at least one pyrethroid pesticide [29]. Thompson et al. [50] compared pesticide urinary metabolite levels of farmworker and non-farmworker Latinx children and adults in the Lower Yakima Valley of Washington. Based on the analysis of the dialkylphosphate urinary metabolite of organophosphate pesticides, they reported that most farmworker and non-farmworker children had been exposed to a pesticide, although the percentage exposed varied reflecting agricultural activities across the year.

The analyses by Harley et al. [23] and Arcury et al. [51] allow a more direct comparison of the measures of pesticide exposure from this current study with those for Latinx children and adolescents living in California and North Carolina agricultural communities (Table 11). These two studies used the same type of wristbands and laboratory analysis to measure pesticide exposure as in the current study. The results of the three studies indicate that each population experiences high frequencies of pesticide exposure, but with different patterns of exposure. The organochlorines DDE, Dicofol, and Endrin aldehyde, were detected for a large proportion of the Californian participants, and a high percentage of the current study participants had detections of alpha-Chlordane, gamma-Chlordane, and trans-Nonachlor. A high percentage of participants in all three studies had detections of several pyrethroids, including Cypermethrin, cis-Permethrin, and trans-Permethrin, with more of the participants in the California study having detections of Esfenvalerate. Participants in the current study had a large percentage of detections of the organophosphate Chlorpyrifos, with a large percentage of detections of Ethion and Ethoprophoa for the participants in the California study. The California study also reported a large percentage of detections of the phenylpyrazole pesticides Fipronil-sulfide and Fipronil-sulfone, the fungicide Chloroneb, and the herbicides Propachlor, Dacthal, and Oxadiazon. These differences partially reflect differences in research design, but also differences in the pesticides used in different areas at different times of the year.

Pesticide exposure is associated with health outcomes. Many of these health effects take years to develop and we cannot document them given the short duration of PACE5. However, our analyses indicate associations of pesticide exposure with child brain structure [52] and cognitive status [53].

### Limitations

This analytic study should be evaluated in terms of its limitations. We did not select participants randomly; we recruited participants from two circumscribed areas of a single state. Our exclusion criteria included neurocognitive conditions that would not allow the participant to complete components of the larger study. These neurocognitive conditions could result from pesticide exposure. However, these exclusion criteria did not affect this analysis, firstly because the neurocognitive conditions would have occurred in advance of our analysis of current pesticide exposure, and secondly because we did not actually exclude any child from the study due to a neurocognitive condition. Current laboratory procedures limit the available measures of pesticide detection and concentration. Our procedures allowed us to measure exposure to a pesticide but not the actual dose of the pesticide that a child might have absorbed. At the same time, our sample is relatively large with cohorts living in diverse environments. We measured a large number of pesticides using laboratory procedures that investigators have applied in a variety of studies [23,41,48,54].

## 5. Conclusions

Pesticides are ubiquitous in the urban as well as rural living environments of Latinx children in vulnerable communities. This analysis adds to current knowledge by documenting the degree to which urban and rural children experience pesticide exposure, and it indicates that the types of pesticide to which children are exposed differ by locality and season. Continuing research must document the pervasive nature of pesticide exposure among children. This research should describe and model the importance of residential locality, as well as region, in terms of the pesticides to which children are exposed. It should also measure seasonal differences in the pesticides to which children are exposed. Research should model the proximate determinants of pesticide exposure in different localities, regions, and seasons to thereby propose ways to intervene in order to reduce child pesticide exposure. Our results suggest that housing quality and time spent in substandard housing is an important determinant of pesticide exposure. We need greater longitudinal research to determine the potential health effects of children’s long-term exposure to the plethora of pesticides in their environments.

We have sufficient data to argue for interventions to reduce child pesticide exposure in all communities, and particularly in vulnerable communities. Although education is important, our efforts must surpass simply teaching families about pesticide safety. Pesticides are endemic in most residential and occupational environments, particularly in those structures and neighborhoods in which vulnerable communities live. Existing pesticides must be removed from these environments. Additional pesticides continue to be applied throughout communities—in dwellings and workplaces, on lawns and in parks, on crops, and on roadways. Our efforts must address approaches to reduce pesticide use. Educating a population to be aware of these applications may be a first step in this process, but effective public health policy will be required to resolve widespread pesticide exposure.

## Figures and Tables

**Table 1:** Baseline Child Characteristics, PACE5 Study, 2018–2022 (*n* = 136).

Child Characteristics	*n* (%)
Locality	
Rural farmworker	75 (55.1)
Urban non-farmworker	61 (44.9)
Gender	
Girl	68 (50.0)
Boy	68 (50.0)
US born	125 (91.9)
Both parents present ^a^	116 (86.6)
Number of people in family ^b^	
3–4	42 (32.3)
5–6	67 (51.5)
7 or more	21 (16.2)

^a^ two missing; ^b^ six missing.

**Table 2:** Pesticide-Exposure Assessments per Season and Pesticide-Exposure Assessments per Child Among Rural Farmworker and Urban Non-Farmworker Children, PACE5 study, 2018–2022.

Exposure Assessments per Season	Rural Farmworker (*n* = 695)	Urban Non-Farmworker (*n* = 473)	Total Sample (*n* = 1168)
	*n* (%)	*n* (%)	*n* (%)
Winter (December–February)	183 (26.3)	113 (23.9)	296 (25.3)
Spring (March–May)	154 (22.2)	81 (17.1)	235 (20.1)
Summer (June–August)	186 (26.8)	144 (30.4)	330 (28.3)
Fall (September–November)	172 (24.7)	135 (28.5)	307 (26.3)
**Exposure Assessments per Child**	**Rural** **Farmworker** ***(n* = 75)**	**Urban** **Non-Farmworker** ***(n* = 61)**	**Total** ***(n* = 136)**
	***n* (%)**	***n* (%)**	***n* (%)**
Number of seasons with an exposure assessment per child			
1	2 (2.7)	6 (9.8)	8 (5.9)
2	2 (2.7)	5 (8.2)	7 (5.1)
3	0 (0.0)	3 (4.9)	3 (2.2)
4	71 (94.7)	47 (77.0)	118 (86.8)
Number of exposure assessments per child			
1	2 (2.7)	6 (9.8)	8 (5.9)
2	1 (1.3)	5 (8.2)	6 (4.4)
3	1 (1.3)	2 (3.3)	3 (2.2)
4	1 (1.3)	1 (1.6)	2 (1.5)
6	0 (0.0)	1 (1.6)	1 (0.7)
8	1 (1.3)	4 (6.6)	5 (3.7)
9	14 (18.7)	11 (18.0)	25 (18.4)
10	55 (73.3)	31 (50.8)	86 (63.2)

**Table 3:** Specific Pesticides and Pesticide Degradation Products Included in Each Class and Their Frequency of Detection by Locality and Season, PACE5 Study, 2018–2022.

Specific Pesticides and Pesticide Degradation Products	Total *n* = 1168	Rural Farmworker *n* = 695	Urban Non-Farmworker *n* = 473	Winter *n* = 296	Spring *n* = 235	Summer *n* = 330	Fall *n* = 307
	*n* (%)	*n* (%)	*n* (%)	*n* (%)	*n* (%)	*n* (%)	*n* (%)
**Insecticides**							
**Organochlorine (25 detected) ^a^**							
4-4-DDD	2 (0.17)	1 (0.1)	1 (0.2)	0 (0.0)	1 (0.4)	0 (0.0)	1 (0.3)
4-4-DDE	89 (7.6)	52 (7.5)	37 (7.8)	20 (6.8)	17 (7.2)	23 (7.0)	29 (9.4)
4-4-DDT	18 (1.5)	11 (1.6)	7 (1.5)	1 (0.3)	4 (1.7)	6 (1.8)	7 (2.3)
Aldrin	21 (1.8)	6 (0.9)	15 (3.2)	5 (1.7)	6 (2.6)	5 (1.5)	5 (1.6)
alpha-Chlordane	839 (71.8)	437 (62.9)	402 (85.0)	243 (82.1)	149 (63.4)	214 (64.8)	233 (75.9)
gamma-Chlordane	874 (74.8)	462 (66.5)	412 (87.1)	245 (82.8)	152 (64.7)	234 (70.9)	243 (79.2)
beta-BHC	4 (0.34)	1 (0.1)	3 (0.6)	1 (0.3)	2 (0.9)	0 (0.0)	1 (0.3)
Chloropropylate	1 (0.09)	0 (0.0)	1 (0.2)	0 (0.0)	1 (0.4)	0 (0.0)	0 (0.0)
Chlorothalonil	59 (5.1)	42 (6.0)	17 (3.6)	18 (6.1)	6 (2.6)	19 (5.8)	16 (5.2)
o,p′-Dicofol	3 (0.3)	1 (0.1)	2 (0.4)	0 (0.0)	2 (0.9)	1 (0.3)	0 (0.0)
p,p′-Dicofol	1 (0.1)	0 (0.0)	1 (0.2)	0 (0.0)	0 (0.0)	0 (0.0)	1 (0.3)
Dieldrin	328 (28.1)	127 (18.3)	201 (42.5)	88 (29.7)	58 (24.7)	78 (23.6)	104 (33.9)
Endosulfan I	41 (3.5)	23 (3.3)	18 (3.8)	6 (2.0)	3 (1.3)	13 (3.9)	19 (6.2)
Endosulfan Sulfate	4 (0.3)	3 (0.4)	1 (0.2)	0 (0.0)	1 (0.4)	1 (0.3)	2 (0.7)
Endrin	16 (1.8)	12 (1.7)	4 (0.8)	6 (2.0)	0 (0.0)	5 (1.5)	5 (1.6)
Endrin aldehyde	4 (0.3)	3 (0.4)	1 (0.2)	2 (0.7)	0 (0.0)	1 (0.3)	1 (0.3)
Heptachlor	134 (11.5)	40 (5.8)	94 (19.9)	44 (14.9)	25 (10.6)	34 (10.3)	31 (10.1)
Heptachlor_epoxide	41 (3.5)	14 (2.0)	27 (5.7)	11 (3.7)	7 (3.0)	14 (4.2)	9 (2.9)
Hexachlorobenzene	3 (0.3)	3 (0.4)	0 (0.0)	2 (0.7)	0 (0.0)	0 (0.0)	1 (0.3)
Isodrin	6 (0.5)	5 (0.7)	1 (0.2)	2 (0.7)	1 (0.4)	1 (0.3)	2 (0.7)
Lindane	11 (0.9)	10 (1.4)	1 (0.2)	2 (0.7)	3 (1.3)	1 (0.3)	5 (1.6)
Methoxychlor	14 (1.2)	10 (1.4)	4 (0.8)	5 (1.7)	2 (0.9)	3 (0.9)	4 (1.3)
Mirex	20 (1.7)	7 (1.0)	13 (2.7)	6 (2.0)	2 (0.9)	6 (1.8)	6 (2.0)
trans-Nonachlor	825 (70.6)	430 (61.9)	395 (83.5)	234 (79.1)	151 (64.3)	208 (63.0)	232 (75.6)
Perthane	4 (0.3)	4 (0.6)	0 (0.0)	2 (0.7)	1 (0.4)	1 (0.3)	0 (0.0)
**Pyrethroid (eight detected)**							
Bifenthrin	27 (2.3)	21 (3.0)	6 (1.3)	3 (1.0)	7 (3.0)	11 (3.3)	6 (2.0)
Cyfluthrin	18 (1.5)	14 (2.0)	4 (0.8)	6 (2.0)	4 (1.7)	4 (1.2)	4 (1.3)
Cypermethrin	761 (65.2)	476 (68.5)	285 (60.3)	212 (71.6)	130 (55.3)	200 (60.6)	219 (71.3)
cis-Permethrin	564 (48.3)	334 (48.1)	230 (48.6)	162 (54.7)	95 (40.4)	156 (47.3)	151 (49.2)
trans-Permethrin	578 (49.5)	338 (48.6)	240 (50.7)	163 (55.1)	98 (41.7)	164 (49.7)	153 (49.8)
deltamethrin and tralomethrin	38 (3.3)	30 (4.3)	8 (1.7)	10 (3.4)	7 (3.0)	12 (3.6)	9 (2.9)
Esfenvalerate	92 (7.9)	50 (7.2)	42 (8.9)	19 (6.4)	21 (8.9)	31 (9.4)	21 (6.8)
L-Cyhalothrin	74 (6.3)	26 (3.7)	48 (10.1)	20 (6.8)	13 (5.5)	22 (6.7)	19 (6.2)
**Organophosphate (nine detected) ^b^**							
Chlorpyrifos	664 (56.9)	456 (65.6)	208 (44.0)	193 (65.2)	128 (54.5)	169 (51.2)	174 (56.7)
Diazinon	1 (0.09)	0 (0.0)	1 (0.2)	0 (0.0)	0 (0.0)	0 (0.0)	1 (0.3)
Dimethoate	5 (0.4)	3 (0.4)	2 (0.4)	0 (0.0)	1 (0.4)	3 (0.9)	1 (0.3)
Ethion	12 (1.0)	5 (0.7)	7 (1.5)	4 (1.4)	2 (0.9)	3 (0.9)	3 (1.0)
Ethoprophos	16 (1.4)	7 (1.0)	9 (1.9)	1 (0.3)	2 (0.9)	6 (1.8)	7 (2.3)
Fenitrothion	2 (0.2)	2 (0.3)	0 (0.0)	0 (0.0)	0 (0.0)	2 (0.6)	0 (0.0)
Imidan	2 (0.2)	2 (0.3)	0 (0.0)	1 (0.3)	0 (0.0)	0 (0.0)	1 (0.3)
Parathion-ethyl	8 (0.9)	7 (1.0)	1 (0.2)	0 (0.0)	6 (2.6)	1 (0.3)	1 (0.3)
Parathion-methyl	1 (0.1)	0 (0.0)	1 (0.2)	0 (0.0)	0 (0.0)	0 (0.0)	1 (0.3)
**Phenylpyrazole (three detected)**							
Fipronil	17 (1.5)	10 (1.4)	7 (1.5)	1 (0.3)	3 (1.3)	7 (2.1)	6 (2.0)
Fipronil-sulfide	151 (12.9)	70 (10.1)	81 (17.1)	35 (11.8)	26 (11.1)	60 (18.2)	30 (9.8)
Fipronil-sulfone	28 (2.4)	16 (2.3)	12 (2.5)	7 (2.4)	9 (3.8)	8 (2.4)	4 (1.3)
Neonicatinoid–acetamiprid	1 (0.5)	0 (0.0)	1 (2.0)	0 (0.0)	1 (1.0)	0 (0.0)	0 (0.0)
**Fungicides**							
Aromatic Fungicide-Chloroneb	141 (12.1)	74 (10.6)	67 (14.2)	38 (12.8)	21 (8.9)	52 (15.8)	30 (9.8)
**Dicarboximide ^c^**							
Captan ^c^	29 (2.5)	18 (2.6)	11 (2.3)	11 (3.7)	11 (4.7)	3 (0.9)	4 (1.3)
Vincolzolin	1 (0.1)	1 (0.1)	0 (0.0)	1 (0.3)	0 (0.0)	0 (0.0)	0 (0.0)
Pentachloronitrobenzene	0						
Thiadiazole-Etridiazole	1 (0.1)	0 (0.0)	1 (0.2)	1 (0.3)	0 (0.0)	0 (0.0)	0 (0.0)
**Herbicides**							
**Dinitroaniline (two detected)**							
Pendimethalin	8 (0.7)	3 (0.4)	5 (1.1)	1 (0.3)	2 (0.9)	4 (1.2)	1 (0.3)
Trifuralin	44 (3.8)	27 (3.9)	17 (3.6)	5 (1.7)	3 (1.3)	14 (4.2)	22 (7.2)
**Aniline/C** **hloroacetanilide (three detected) ^d^**							
Alachlor	2 (0.2)	1 (0.1)	1 (0.2)	0 (0.0)	1 (0.4)	1 (0.3)	0 (0.0)
Metolachlor	14 (1.2)	8 (1.2)	6 (1.3)	4 (1.4)	2 (0.9)	6 (1.8)	2 (0.7)
Propachlor	33 (2.8)	19 (2.7)	14 (3.0)	5 (1.7)	6 (2.6)	12 (3.6)	10 (3.3)
**Triazine (two detected)**							
Atrazine	1 (0.1)	1 (0.1)	0 (0.0)	0 (0.0)	1 (0.4)	0 (0.0)	0 (0.0)
Simazine	6 (2.0)	5 (2.6)	1 (0.9)	0 (0.0)	3 (2.9)	3 (3.3)	0 (0.0)
**Benzenedicarboxylic acid** *—* **Dacthal**	4 (0.3)	4 (0.6)	0 (0.0)	0 (0.0)	1 (0.4)	3 (0.9)	0 (0.0)
**Oxadiazole-Oxadiazon**	12 (1.0)	5 (0.7)	7 (1.5)	2 (0.7)	5 (2.1)	3 (0.9)	2 (0.7)
**Thiocarbamate-Diallate I**	2 (0.2)	1 (0.1)	1 (0.2)	0 (0.0)	1 (0.4)	1 (0.3)	0 (0.0)

^a^ Organochlorine pesticides not detected: alpha-BHC; Chlorobenzilate; delta-BHC; Endosulfan II; Endrin Ketone; ^b^ Organophosphate pesticides not detected: Chlorpyrifos_Methyl; Fonofos; Phorate; ^c^ Dicarboximide pesticides not detected: Captafol; Iprodione; ^d^ Aniline/Chloroacetanilide pesticides not detected: Propanil.

**Table 4:** Main Effect Models for Detection Differences by Locality and Season for Commonly Detected Pesticides and Pesticide Degradation Products (at least 10% detections), PACE5 Study, Total Sample (*n* = 1168), 2018–2022.

Specific Pesticides and Pesticide Degradation Products	Rural Farmworker versus Urban Non-Farmworker		Spring versus Winter		Summer versus Winter		Fall versus Winter	
	Odds Ratio (95% CI ^a^)	*p*-Value	Odds Ratio (95% CI ^a^)	*p*-Value	Odds Ratio (95% CI ^a^)	*p*-Value	Odds Ratio (95% CI ^a^)	*p*-Value
**Organochlorines**								
alpha-Chlordane	0.26 (0.17, 0.41)	<0.0001	0.38 (0.26, 0.57)	<0.0001	0.35 (0.24, 0.51)	<0.0001	0.62 (0.44, 0.89)	0.0092
gamma-Chlordane	0.27 (0.17, 0.42)	<0.0001	0.37 (0.25, 0.55)	<0.0001	0.45 (0.31, 0.66)	<0.0001	0.73 (0.50, 1.06)	0.1007
Dieldrin	0.29 (0.19, 0.44)	<0.0001	0.79 (0.55, 1.14)	0.2154	0.67 (0.47, 0.96)	0.0277	1.15 (0.85, 1.55)	0.3609
trans-Nonachlor ^b^	0.29 (0.18, 0.45)	<0.0001	0.48 (0.33, 0.68)	<0.0001	0.39 (0.27, 0.57)	<0.0001	0.76 (0.55, 1.04)	0.0880
**Pyrethroids**								
Cypermethrin	1.48 (0.98, 2.24)	0.0649	0.53 (0.39, 0.72)	<0.0001	0.65 (0.49, 0.87)	0.0033	1.02 (0.76, 1.36)	0.8881
cis-Permethrin ^b^	0.99 (0.69, 1.42)	0.9613	0.56 (0.43, 0.74)	<0.0001	0.76 (0.57, 1.01)	0.0564	0.82 (0.63, 1.08)	0.1534
trans-Permethrin ^b^	0.93 (0.65, 1.34)	0.7067	0.59 (0.44, 0.78)	0.0003	0.82 (0.62, 1.09)	0.1705	0.83 (0.64, 1.09)	0.1773
**Organophosphates**								
Chlorpyrifos	2.43 (1.62, 3.62)	<0.0001	0.63 (0.46, 0.86)	0.0036	0.61 (0.47, 0.79)	0.0002	0.75 (0.55, 1.02)	0.0639
**Phenylpyrazole**								
Fipronil-sulfide ^b^	0.54 (0.36, 0.81)	0.0031	0.98 (0.58, 1.65)	0.9430	1.71 (1.09, 2.70)	0.0205	0.80 (0.49, 1.30)	0.3687
**Aromatic Fungicide**								
Chloroneb	0.74 (0.50, 1.07)	0.1111	0.67 (0.37, 1.22)	0.1913	1.24 (0.82, 1.90)	0.3102	0.72 (0.45, 1.17)	0.1890

^a^ 95% CI: 95% confidence interval; ^b^ Significant locality by season interaction.

**Table 5:** Significant Locality–Season Interactions for Detections of Commonly Detected Pesticides and Pesticide Degradation Products, PACE5 Study, Total Sample (*n* = 1168), 2018–2022.

Season	Locality		Interaction		
	Urban Non-Farmworker	Rural Farmworker			
Pesticide	Probability (95% CI ^a^)	Probability (95%CI ^a^)	Odds Ratio (95%CI ^a^)	*p*-Value	*p* for Interaction
trans-Nonachlor					
Winter	0.87 (0.77, 0.93)	0.75 (0.68, 0.80)	0.46 (0.22, 0.97)	0.0404	0.0136
Spring	0.90 (0.80, 0.95)	0.51 (0.44, 0.59)	0.12 (0.05, 0.28)	<.0001	
Summer	0.76 (0.68, 0.84)	0.52 (0.45, 0.60)	0.34 (0.20, 0.59)	0.0001	
Fall	0.86 (0.79, 0.92)	0.68 (0.61, 0.74)	0.33 (0.17, 0.61)	0.0004	
cis-Permethrin					
Winter	0.47 (0.37, 0.56)	0.59 (0.52, 0.66)	1.67 (1.03, 2.71)	0.0393	0.0127
Spring	0.44 (0.33, 0.57)	0.38 (0.30, 0.46)	0.76 (0.42, 1.37)	0.3587	
Summer	0.52 (0.43, 0.61)	0.44 (0.37, 0.51)	0.72 (0.45, 1.17)	0.1888	
Fall	0.49 (0.40, 0.58)	0.50 (0.42, 0.58)	1.03 (0.63, 1.68)	0.9084	
trans-Permethrin					
Winter	0.49 (0.40, 0.58)	0.58 (0.51, 0.65)	1.43 (0.89, 2.31)	0.1408	0.0387
Spring	0.45 (0.33, 0.58)	0.39 (0.32, 0.47)	0.78 (0.43, 1.40)	0.4064	
Summer	0.55 (0.46, 0.64)	0.46 (0.38, 0.53)	0.67 (0.42, 1.08)	0.0984	
Fall	0.50 (0.41, 0.59)	0.50 (0.43, 0.58)	1.02 (0.63, 1.63)	0.9451	
Fipronil-sulfide					
Winter	0.22 (0.16, 0.30)	0.05 (0.02, 0.11)	0.18 (0.07, 0.46)	0.0003	0.0001
Spring	0.06 (0.03, 0.14)	0.14 (0.10, 0.20)	2.48 (0.93, 6.61)	0.0690	
Summer	0.23 (0.16, 0.31)	0.16 (0.12, 0.21)	0.64 (0.37, 1.10)	0.1074	
Fall	0.14 (0.09, 0.22)	0.06 (0.03, 0.11)	0.41 (0.18, 0.91)	0.0295	

^a^ 95% CI: 95% confidence interval.

**Table 6:** Concentration ^a^ (ng/g) Differences by Locality and Season for Commonly Detected Pesticides and Pesticide Degradation Products (at least 10% detections), PACE5 Study, Total Sample (*n* = 1168), 2018–2022.

Specific Pesticides and Pesticide Degradation Products	Concentration			
	Log Mean (SE ^b^)	Geometric Mean (95% CI ^c^)	*p*-Value	
**Organochlorines**				
alpha-Chlordane				
Locality			0.0013	
Rural farmworker	2.08 (0.10)	7.99 (6.56, 9.73)		
Urban non-farmworker	2.57 (0.11)	13.02 (10.48, 16.18)		
Season				
Winter	2.19 (0.08)	8.95 (7.60, 10.54)	0.0003	
Spring	2.26 (0.09)	9.54 (7.99, 11.38)		
Summer	2.45 (0.08)	11.54 (9.77, 13.64)		
Fall	2.40 (0.08)	10.98 (9.32, 12.93)		
gamma-Chlordane				
Locality			0.0004	
Rural farmworker	1.97 (0.10)	7.14 (5.84, 8.74)		
Urban non-farmworker	2.52 (0.11)	12.46 (9.96, 15.58)		
Season				
Winter	2.20 (0.09)	9.02 (7.60, 10.72)	0.0016	
Spring	2.08 (0.10)	7.98 (6.61, 9.64)		
Summer	2.35 (0.09)	10.53 (8.85, 12.53)		
Fall	2.35 (0.09)	10.44 (8.79, 12.40)		
Dieldrin				
Locality			0.0002	
Rural farmworker	1.97 (0.11)	7.16 (5.78, 8.86)		
Urban non-farmworker	2.56 (0.11)	12.97 (10.46, 16.08)		
Season			0.0711	
Winter	2.11 (0.10)	8.26 (6.81, 10.02)		
Spring	2.27 (0.11)	9.70 (7.78, 12.10)		
Summer	2.39 (0.10)	10.90 (8.91, 13.34)		
Fall	2.29 (0.09)	9.88 (8.24, 11.84)		
trans-Nonachlor				
Locality			0.0061	
Rural farmworker	1.34 (0.10)	3.84 (3.16, 4.67)		
Urban non-farmworker	1.76 (0.11)	5.79 (4.66, 7.20)		
Season			<0.0001	
Winter	1.48 (0.08)	4.41 (3.74, 5.21)		
Spring	1.34 (0.09)	3.83 (3.20, 4.58)		
Summer	1.70 (0.09)	5.47 (4.62, 6.49)		
Fall	1.68 (0.08)	5.35 (4.53, 6.32)		
**Pyrethroids**				
Cypermethrin				
Locality			<0.0001	
Rural farmworker	5.76 (0.13)	316.36 (244.39, 409.51)		
Urban non-farmworker	4.87 (0.16)	129.73 (94.97, 177.19)		
Season			0.0055	
Winter	5.28 (0.12)	196.67 (154.42, 250.49)		
Spring	5.06 (0.14)	157.07 (119.49, 206.47)		
Summer	5.52 (0.13)	249.90 (195.28, 319.79)		
Fall	5.39 (0.12)	218.17 (171.50, 277.55)		
cis-Permethrin				
Locality			0.0505	
Rural farmworker	5.09 (0.10)	162.68 (132.31, 200.02)		
Urban non-farmworker	4.77 (0.13)	118.02 (92.12, 151.22)		
Season			0.0017	
Winter	4.83 (0.11)	125.23 (100.84, 155.52)		
Spring	4.67 (0.13)	106.72 (81.90, 139.07)		
Summer	5.20 (0.11)	180.73 (145.04, 225.19)		
Fall	5.03 (0.11)	152.62 (122.48, 190.19)		
trans-Permethrin				
Locality			0.0426	
Rural farmworker	5.69 (0.10)	294.71 (240.55, 361.07)		
Urban non-farmworker	5.36 (0.12)	213.18 (167.78, 270.86)		
Season			0.0572	
Winter	5.42 (0.11)	226.63 (183.34, 280.13)		
Spring	5.38 (0.13)	217.78 (168.71, 281.12)		
Summer	5.71 (0.11)	300.48 (243.40, 370.94)		
Fall	5.58 (0.11)	266.15 (214.51, 330.23)		
**Organophosphates**				
Chlorpyrifos				
Locality			0.0107	
Rural farmworker	2.56 (0.11)	12.91 (10.39, 16.04)		
Urban non-farmworker	2.10 (0.14)	8.16 (6.20, 10.75)		
Season			0.0042	
Winter	2.18 (0.10)	8.83 (7.21, 10.80)		
Spring	2.27 (0.11)	9.70 (7.80, 12.07)		
Summer	2.48 (0.10)	11.98 (9.76, 14.70)		
Fall	2.38 (0.10)	10.83 (8.83, 13.28)		
**Phenylpyrazole**				
Fipronil-sulfide				
Locality			0.6018	
Rural farmworker	4.20 (0.16)	66.77 (48.45, 92.01)		
Urban non-farmworker	4.08 (0.16)	59.26 (43.04, 81.60)		
Season			0.2437	
Winter	3.85 (0.19)	47.20 (32.31, 68.95)		
Spring	4.34 (0.22)	76.41 (49.65, 117.59)		
Summer	4.27 (0.14)	71.66 (53.83, 95.40)		
Fall	4.10 (0.20)	60.58 (40.57, 90.46)		
**Aromatic fungicide**				
Chloroneb				
Locality			0.3755	
Rural farmworker	4.49 (0.17)	88.92 (63.67, 124.19)		
Urban non-farmworker	4.27 (0.18)	71.52 (49.91, 102.48)		
Season			0.6751	
Winter	4.52 (0.19)	91.96 (63.62, 132.90)		
Spring	4.24 (0.25)	69.62 (42.66, 113.63)		
Summer	4.30 (0.16)	73.35 (53.02, 101.47)		
Fall	4.46 (0.21)	86.12 (57.27, 129.50)		

^a^ Values above the limit of detection; ^b^ SE: standard error; ^c^ 95% CI: 95% confidence interval.

**Table 7:** Detection Differences for Pesticide Classes for Locality and Season, PACE5 Study, Total Sample (*n* = 1168), 2018–2022.

Number of Detections of at Least One Pesticide Class	Total *n* = 1168	Rural Farmworker *n* = 695	Urban Non-Farmworker *n* = 473	Winter *n* = 296	Spring *n* = 235	Summer *n* = 330	Fall *n* = 307
	*n* (%)	*n* (%)	*n* (%)	*n* (%)	*n* (%)	*n* (%)	*n* (%)
**Insecticides**							
Organochlorine	996 (85.3)	552 (79.4)	444(93.9)	263 (88.9)	187 (79.6)	272 (82.4)	274 (89.3)
Pyrethroid	906 (77.6)	532(76.5)	374 (79.1)	240 (81.1)	156 (66.4)	255 (77.3)	255 (83.1)
Organophosphate	688 (58.9)	464 (66.8)	224 (47.4)	194 (65.5)	134 (57.0)	179 (54.2)	181 (59.0)
Phenylpyrazole	185 (15.8)	90 (12.9)	95 (20.1)	40 (13.5)	35 (14.9)	71 (21.5)	39 (12.7)
Neonicatinoid	1 (0.5)	0	1 (.2)	0 (0.0)	1 (1.0)	0 (0.0)	0 (0.0)
**Fungicides**							
Aromic fungicide (Chloroneb)	141 (12.1)	74 (10.6)	67 (14.2)	38 (12.8)	21 (8.9)	52 (15.8)	30 (9.8)
Dicarboximide	30 (2.6)	19 (2.7)	11 (2.3)	12 (4.1)	11 (4.7)	3 (0.9)	4 (1.3)
Pentachloronitrobenzene	0						
Thiadiazole	1 (0.1)	0 (0.0)	1 (0.2)	1 (0.3)	0 (0.0)	0 (0.0)	0 (0.0)
**Herbicides**							
Dinitroaniline	52 (4.5)	30 (4.3)	22 (4.7)	6 (2.0)	5 (2.1)	18 (5.5)	23 (7.5)
Aniline/Chloroacetanilide	47 (4.0)	28 (4.0)	19 (4.0)	9 (3.0)	8 (3.4)	19 (5.8)	11 (3.6)
Triazine	7 (0.6)	6 (0.9)	1 (0.2)	0 (0.0)	4 (1.7)	3 (0.9)	0 (0.0)
Benzenedicarboxylic acid	4 (0.3)	4 (0.6)	0 (0.0)	0 (0.0)	1 (0.4)	3 (0.9)	0 (0.0)
Oxadiazole	12 (1.0)	5 (0.7)	7 (1.5)	2 (0.7)	5 (2.1)	3 (0.9)	2 (0.7)
Thiocarbamate	2 (0.2)	1 (0.1)	1 (0.2)	0 (0.0)	1 (0.4)	1 (0.3)	0 (0.0)

**Table 8:** Main Effect Models for Detection Differences by Locality and Season for Commonly Detected Pesticide Classes (at least 10% detections), PACE5 Study, Total Sample (*n* = 1168), 2018–2022.

Commonly Detected Pesticide Classes	Rural Farmworker versus Urban Non-Farmworker		Spring versus Winter		Summer versus Winter		Fall versus Winter	
	Odds Ratio (95% CI ^a^)	*p*-Value	Odds Ratio (95% CI ^a^)	*p*-Value	Odds Ratio (95% CI ^a^)	*p*-Value	Odds Ratio (95% CI ^a^)	*p*-Value
Any Organochlorine detection	0.25 (0.14, 0.43)	<.0001	0.52 (0.33, 0.84)	0.0078	0.57 (0.37, 0.88)	0.0121	0.97 (0.62, 1.50)	0.8838
Any Pyrethroid detection	0.88 (0.59, 1.33)	0.5474	0.49 (0.34, 0.69)	<.0001	0.81 (0.56, 1.16)	0.2526	1.14 (0.79, 1.63)	0.4881
Any Organophosphate detection	2.21 (1.49, 3.29)	<.0001	0.69 (0.51, 0.93)	0.0142	0.67 (0.52, 0.88)	0.0034	0.81 (0.59, 1.10)	0.1796
Any Phenylpyrazole detection ^b^	0.60 (0.42, 0.84)	0.0036	1.16 (0.73, 1.86)	0.5209	1.76 (1.15, 2.69)	0.0088	0.92 (0.59, 1.43)	0.7009
Any Chloroneb detection	0.74 (0.50, 1.07)	0.1111	0.67 (0.37, 1.22)	0.1913	1.24 (0.82, 1.90)	0.3102	0.72 (0.45, 1.17)	0.1890

^a^ 95% CI: 95% confidence interval; ^b^ Significant locality–season interaction.

**Table 9:** Significant Locality–Season Interactions for Detections of Pesticide Classes, PACE5 Study, Total Sample (*n* = 1168), 2018–2022.

Season	Locality		Interaction		
	Urban Non-Farmworker	Rural Farmworker			
Pesticide Class	Probability (95% CI ^a^)	Probability (95%CI ^a^)	Odds Ratio (95%CI ^a^)	*p*-Value	*p* for Interaction
Phenylpyrazole					
Winter	0.23 (0.17, 0.31)	0.07 (0.04, 0.13)	0.25 (0.11, 0.53)	0.0004	0.0067
Spring	0.12 (0.07, 0.20)	0.16 (0.12, 0.22)	1.41 (0.69, 2.88)	0.3475	0.0067
Summer	0.26 (0.19, 0.34)	0.19 (0.15, 0.24)	0.68 (0.40, 1.13)	0.1367	0.0067
Fall	0.16 (0.11, 0.24)	0.10 (0.06, 0.15)	0.56 (0.28, 1.10)	0.0900	0.0067

^a^ 95% CI: 95% confidence interval.

**Table 10:** Average Detection Differences for Total Class, Total Specific Pesticides, Number of Organochlorine, and Number of Pyrethroid Detections by Locality and Season, PACE5 Study, Total Sample (*n* = 1168), 2018–2022.

Average Total Specific Pesticides, Number of Organochlorine, and Number of Pyrethroid Detections	Total *n* = 1168	Rural Farmworker *n* = 695	Urban Non-Farmworker *n* = 473		Winter *n* = 296	Spring *n* = 235	Summer *n* = 330	Fall *n* = 307	
	Mean (SD ^a^)	Mean (SE ^b^)	Mean (SE ^b^)	*p*-Value	Mean (SE ^b^)	Mean (SE ^b^)	Mean (SE ^b^)	Mean (SE ^b^)	*p*-Value
Total pesticide class detections	2.6 (1.2)	2.6 (0.06)	2.7 (0.07)	0.4628	2.7 (0.07)	2.4 (0.08)	2.7 (0.07)	2.7 (0.07)	0.0209
Total specific pesticide detections	5.8 (2.8)	5.4 (0.16)	6.3 (0.19)	0.0003	6.4 (0.18)	5.3 (0.19)	5.6 (0.17)	6.1 (0.17)	<0.0001
Number of Organochlorine detections	2.9 (1.7)	2.5 (0.09)	3.5 (0.11)	<0.0001	3.3 (0.10)	2.7 (0.11)	2.7 (0.10)	3.2 (0.10)	<0.0001
Number of Pyrethroid detections	1.8 (1.4)	1.8 (0.08)	1.8 (0.10)	0.7231	2.0 (0.09)	1.6 (0.10)	1.8 (0.09)	1.9 0.093)	0.0032

^a^ SD: standard deviation; ^b^ SE: standard error.

**Table 11:** Percent detections of frequently detected pesticides (> 15% in at least one study) comparing results from Harley et al., 2019 and Arcury et al., 2021b with those of the current study.

Pesticide	Harley et al. [23]	Arcury et al. [51]		Current Study		
	California	North Carolina		North Carolina		
	Percent Detections for 2016	Percent Detections for 2018	Percent Detections for 2019	Percent Detections Total	Percent Detections Rural Farmworker	Percent Detections Urban Non-Farmworker
**Organochlorines**						
DDE	55.7	1.7	3.8	7.6	.5	7.8
alpha-Chlordane	12.3	22.5	30.1	71.8	62.9	85.0
gamma-Chlordane	9.4	23.7	30.8	74.8	66.5	87.1
Dieldrin	22.7	2.9	5.1	28.1	18.3	42.5
Dicofol	33.0	ND ^a^	0.6	0.3	0.1	0.6
Endrin aldehyde	21.6	3.5	0.0	0.3	0.4	0.2
trans-Nonachlor	14.4	15.0	16.7	70.6	61.9	83.5
**Pyrethroids**						
Cypermethrin	55.7	48.6	47.4	65.2	68.5	60.3
Esfenvalerate	41.2	7.5	3.8	7.9	7.2	8.9
cis-Permethrin	48.5	47.4	24.4	48.3	48.1	48.6
trans-Permethrin	51.5	49.7	27.6	49.5	48.6	50.7
**Organophosphates**						
Chlorpyrifos	36.1	38.7	30.8	56.9	65.6	44.0
Ethion	39.2	2.9	1.9	1.0	0.7	1.5
Ethoprophos	34.0	4.0	0.6	1.4	1.0	1.9
**Phenylpyrazoles**						
Fipronil-sulfide	86.6	4.0	3.2	12.9	10.1	17.1
Fipronil-sulfone	45.4	1.7	0.6	2.4	2.3	2.5
**Fungicides**						
Chloroneb	34.0	3.5	14.1	12.1	10.6	14.2
**Herbicides**						
Propachlor	53.6	11.0	1.9	2.8	2.7	3.0
Dacthal	52.6	2.3	ND	0.3	0.6	0.0
Oxadiazon	21.6	0.6	ND	1.0	0.7	1.5

^a^ ND: not detected.

## Data Availability

The data presented in this study are available on request from the corresponding author. The data are not publicly available to protect participant confidentiality.

## Supplementary Materials

The following supporting information can be downloaded at: https://www.mdpi.com/article/10.3390/ijerph20095647/s1, Table S1: Pesticide analyte list, detection method, and quantitation limits supplement; Table S2: Gas chromatograph–micro electron capture detector for the analysis of pesticides.
