# Supplementary material for: Pesticide Exposure among Latinx Children in Rural Farmworker and Urban Non-Farmworker Communities: Associations with Locality and Season
Source: Int J Environ Res Public Health. 2023 Apr 26;20(9):5647. doi: 10.3390/ijerph20095647 (PMC10178580; doi:10.3390/ijerph20095647)
Supplement: Supplementary file 1 [file ijerph-20-05647-s001.zip › ijerph-2227902-supplementary.pdf]

Supplementary Materials

Table S1. Pesticide Analyte List, Detection Method, and Quantitation Limits.

Common Name	CAS #	RT (DB-17MS)	RT (DB-5MS)	MDL (pg/uL)	MQL (pg/uL)
4,4'-dibromooctafluorobiphenyl	10386-84-2	5.52	5.81	Not Applicable	
TCMX	877-09-8	5.79	5.24	0.95	7.60
PCB 100	39485-83-1	12.19	10.64	4.76	38.07
decachlorobiphenyl*	2051-24-3	19.44	18.64	3.08	24.65
etridiazole*	2593-15-9	4.38	3.63	2.95	23.56
chloroneb	2675-77-6	5.31	4.10	9.86	78.88
trifluralin	1582-09-8	5.38	5.76	3.03	24.22
propachlor	1918-16-7	6.60	5.11	5.72	45.77
prophos*	13194-48-4	6.65	5.42	22.23	177.81
diallate*	2303-16-4	6.88	6.09	20.90	167.17
hexachlorobenzene*	118-74-1	7.24	6.23	0.65	5.16
phorate	298-02-2	7.44	6.09	8.29	66.34
a-BHC*	319-84-6	7.75	6.18	0.37	2.92
pentachloronitrobenzene*	82-68-8	8.52	6.82	1.84	14.76
diazinon*	333-41-5	8.56	7.34	33.07	264.59
atrazine*	1912-24-9	8.76	6.82	13.25	105.98
simazine*	122-34-9	8.94	6.68	21.08	168.63
lindane	58-89-9	8.94	6.96	0.15	1.23
dyfonate	944-22-9	9.03	7.19	6.38	51.01
dimethoate*	60-51-5	9.74	6.48	1.78	14.27
b-BHC*	319-85-7	9.68	6.75	0.80	6.40
heptachlor*	76-44-8	9.78	8.81	2.51	20.07
vinclozolin	50471-44-8	10.09	8.61	38.53	308.23
alachlor	15972-60-8	10.48	8.70	4.96	39.65
d-BHC	319-86-8	10.55	7.67	0.60	4.82
chlorothalonil	1897-45-6	10.62	7.34	3.46	27.69
aldrin*	309-00-2	10.70	9.76	9.88	79.04
methyl chlorpyrifos	5598-13-0	10.75	8.48	1.40	11.17
propanil	709-98-8	10.95	8.37	10.81	86.52
methyl parathion	298-00-0	11.26	8.64	11.87	94.97
metolachlor*	51218-45-2	11.46	9.69	39.05	312.40
o,p'-dicofol	10606-46-9	11.64	9.29	10.50	83.99
dacthal	1861-32-1	11.64	9.90	1.34	10.68
chlorpyrifos	2921-88-2	11.75	9.80	1.82	14.58
fipronil sulfide	120067-83-6	11.91	10.64	3.09	24.75
fenitrothion*	122-14-5	12.02	9.36	2.37	18.96
fipronil	120068-37-3	12.01	10.92	4.36	34.90
isodrin	465-73-6	12.04	10.55	0.30	2.44
parathion	56-38-2	12.14	10.02	6.44	51.51
p,p'-dicofol	115-32-2	12.41	10.19	20.86	166.89
heptachlor epoxide	1024-57-3	12.55	10.86	0.52	4.18
pendimethalin	40487-42-1	12.61	10.72	6.61	52.92
trans-chlordane*	5103-74-2	13.15	11.53	0.99	7.94
trans-nonachlor*	39765-80-5	13.20	11.98	1.08	8.62
cis-chlordane*	5103-71-9	13.55	11.88	1.02	8.18
endosulfan I	959-98-8	13.64	11.88	0.38	3.06
fipronil sulfone	120068-36-2	14.06	12.64	2.93	23.46
oxadiazon*	19666-30-9	14.17	12.78	2.95	23.57
p,p'-DDE*	72-55-9	14.51	12.64	1.46	11.72
dieldrin	60-57-1	14.59	12.64	0.55	4.41
captan	133-06-2	14.88	11.15	41.12	328.95
chloropropylate	005836-10-2	15.14	13.63	5.58	44.62

perthan	72-56-0	15.47	13.45	58.68	469.45
chlorobenzilate	510-15-6	15.55	13.63	10.54	84.33
endrin*	72-70-8	15.61	13.22	2.13	17.04
p,p'-DDD	72-54-8	16.16	13.83	3.56	28.48
ethion*	563-12-2	16.21	13.94	5.23	41.87
endosulfan II	33213-65-9	16.25	13.56	1.09	8.74
p,p'-DDT	50-29-3	16.70	14.92	1.01	8.07
endrin aldehyde	7421-93-4	16.84	13.99	0.44	3.51
bifenthrin	82657-04-3	16.95	16.31	3.31	26.44
endosulfan sulfate	1031-07-8	17.12	14.68	1.46	11.70
iprodione	36734-19-7	17.43	13.73	11.96	95.70
L-cyhalothrin	91465-08-6	17.87	17.241	10.68	85.42
captofol*	002425-06-1	17.95	15.476	12.13	97.05
methoxychlor*	72-43-5	18.021	16.36	2.73	21.80
mirex*	2385-85-5	18.089	17.081	1.48	11.83
endrin ketone*	53494-70-5	18.143	15.895	1.83	14.68
imidan	732-11-6	18.454	16.075	1.55	12.40
cis-permethrin	61949-76-6	18.761	17.818	0.73	5.84
trans-permethrin	61949-77-7	18.848	17.915	1.27	10.13
acetamiprid	135410-20-7	18.99	15.893	11.70	93.61
cyfluthrin	68359-37-5	19.138	18.347	2.54	20.32
cypermethrin	52315-07-8	19.414	18.552	5.08	40.62
esfenvalerate	66230-04-4	20.034	19.093	6.11	48.89
deltamethrin + tralomethrin	52918-63-5 + 66841-25-6	20.505	19.403	5.50	44.01

Chemical Abstract Service (CAS), Retention Times (RT), Method Detection Limits (MDL) and Method Quantitation Limits (MQL)
*-indicates that the LOD was calculated from the study of a 50ppb standard. Both a 10 and a 50ppb standard was ran 7 times sequentially to determine instrument detection limits (IDL), the 50ppb was used when the relative percent error was greater than 20%. IDL was calculated by multiplying the standard deviation by the student T value for the 99% confidence interval (2.998). MDLs are based on the difference in instrument response of an over-spiked wristband extract to a standard. MDLs were calculated by multiplying the IDL by 3 and then by 8 for the MQL.

Table S2. Gas Chormatograph-Micro electron capture Detector for the Analysis of Pesticides.

<table>
<tr><th colspan="4">Instrument</th></tr>
<tr><td colspan="4">Agilent 6890N Gas Chromatograph with dual Agilent Micro ECD's</td></tr>
<tr><th colspan="4">Carrier Gas</th></tr>
<tr><td colspan="4">Hydrogen (99.99%)</td></tr>
<tr><th colspan="4">Columns</th></tr>
<tr><td colspan="2">J&W DB-17MS, 30m, ID 0.25mm, Film 0.25µM</td><td colspan="2">J&W DB-5MS, 30m, ID 0.25mm, Film 0.25µM</td></tr>
<tr><th colspan="4">Inlet Parameters (2µL splitless injection)</th></tr>
<tr><th>Temperature (°C)</th><td>250</td><th>Flow (Mode: Constant Flow)</th><td>2.6 mL/min</td></tr>
<tr><th colspan="4">Oven Program</th></tr>
<tr><th colspan="4">Temperature (°C) and Time (min) program</th></tr>
<tr><td colspan="4">110°C, 0.5 min hold; 25°C/min to 150°C; 6°C to 229°C: 20°C/min to 320°C; 320°C, hold for 2.5 min</td></tr>
<tr><td colspan="4">22.32 min Total Run Time</td></tr>
<tr><th colspan="4">Detectors</th></tr>
<tr><th colspan="2">Temperature (°C)</th><th colspan="2">make-up gas Flow rate (N_2)</th></tr>
<tr><td colspan="2">320</td><td colspan="2">40 mL/min</td></tr>
</table>
